# Supplementary material for: Development of a SFTSV DNA vaccine that confers complete protection against lethal infection in ferrets
Source: Nat Commun. 2019 Aug 23;10:3836. doi: 10.1038/s41467-019-11815-4 (PMC6707330; doi:10.1038/s41467-019-11815-4)
Supplement: Supplementary file 1 — Supplementary Information [file 41467_2019_11815_MOESM1_ESM.pdf]

Supplementary Information

Development of a SFTSV DNA vaccine that confers complete protection against lethal infection in ferrets

Kwak et al.

Contents:

Supplementary Figures 1 – 10
Supplementary Table 1

Supplementary Figure 1

a Mouse study

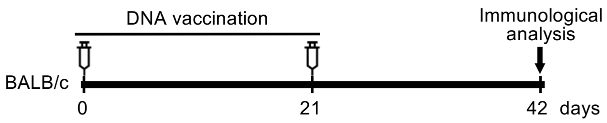

Group		Vaccination		
Control Gr (n=4)	pVax1	(200 µg)	–	
Vaccine Gr (n=4)	pVax1-Gn<br>pVax1-Gc<br>pVax1-N<br>pVax1-NSs<br>pVax1-RdRp	(40 µg)<br>(40 µg)<br>(40 µg)<br>(40 µg)<br>(40 µg)	–	
Vaccine/EP Gr (n=4)	pVax1-Gn<br>pVax1-Gc<br>pVax1-N<br>pVax1-NSs<br>pVax1-RdRp	(40 µg)<br>(40 µg)<br>(40 µg)<br>(40 µg)<br>(40 µg)	+ *in vivo* electroporation	

b Ferret study #1

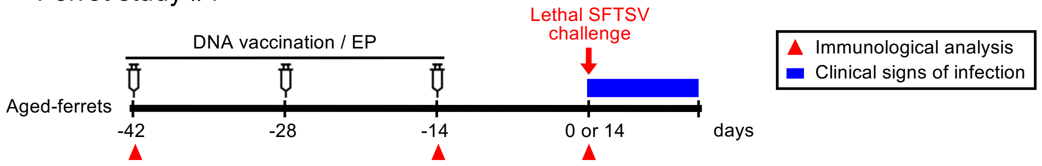

Group	Ferret #	Vaccination		
Control Gr (n=7)	C1-C7	pVax1	(1 mg)	C1-C4, challenge at day 0;<br>C5-C7, challenge at day 14
Vaccine Gr (n=8)	V1-V8	pVax1-Gn<br>pVax1-Gc<br>pVax1-N<br>pVax1-NSs<br>pVax1-RdRp	(200 µg)<br>(200 µg)<br>(200 µg)<br>(200 µg)<br>(200 µg)	V1-V4, challenge at day 0;<br>V5-V8, challenge at day 14
Vaccine 200 µg total Gr (n=3)	V15-V17	pVax1-Gn<br>pVax1-Gc<br>pVax1-N<br>pVax1-NSs<br>pVax1-RdRp	(40 µg)<br>(40 µg)<br>(40 µg)<br>(40 µg)<br>(40 µg)	challenge at day 14
Vaccine 40 µg total Gr (n=3)	V18-V20	pVax1-Gn<br>pVax1-Gc<br>pVax1-N<br>pVax1-NSs<br>pVax1-RdRp	(8 µg)<br>(8 µg)<br>(8 µg)<br>(8 µg)<br>(8 µg)	challenge at day 14

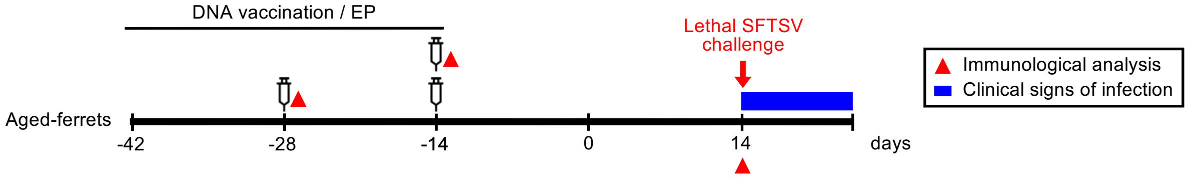

Group	Ferret #	Vaccination		
2 times vaccine Gr (n=3)	V9-V11	pVax1-Gn<br>pVax1-Gc<br>pVax1-N<br>pVax1-NSs<br>pVax1-RdRp	(200 µg)<br>(200 µg)<br>(200 µg)<br>(200 µg)<br>(200 µg)	challenge at day 14
1 time vaccine Gr (n=3)	V12-V14			

Supplementary Figure 1 (Cont.)

c Ferret study #2

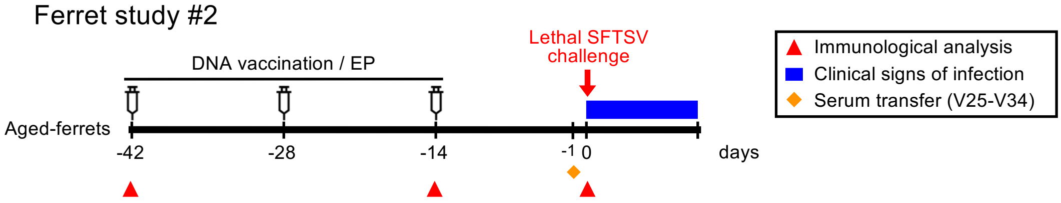

Group	Ferret #	Vaccination
Control Gr (n=6)	C8-C13	pVax1 (1 mg)
Gn/Gc vaccine Gr (n=4)	V21-V24	pVax1-Gn (200 µg)<br>pVax1-Gc (200 µg)
Control serum transfer Gr (n=3)	V25-V27	– transfer of serum at day -1 collected from pVax1-vaccinated ferrets (S1-S2)
Gn/Gc serum transfer Gr (n=4)	V28-V31	– transfer of serum at day -1 collected from Gn/Gc-vaccinated ferrets (S3-S4)
N/NSs/RdRp serum transfer Gr (n=3)	V32-V34	– transfer of serum at day -1 collected from N/NSs/RdRp-vaccinated ferrets (S5-S6)
N/NSs/RdRp vaccine Gr (n=4)	V35-V38	pVax1-N (200 µg)<br>pVax1-NSs (200 µg)<br>pVax1-RdRp (200 µg)
N vaccine Gr (n=3)	V39-V41	pVax1-N (200 µg)
NSs vaccine Gr (n=3)	V42-V44	pVax1-NSs (200 µg)
RdRp vaccine Gr (n=3)	V45-V47	pVax1-RdRp (200 µg)

Supplementary Figure 1. Designs of animal studies. a Timeline of vaccination and immune analysis for a mouse study. BALB/c mice (*n* = 4) were immunized via intramuscular injection with a mixture of SFTSV DNA plasmids (pVax1-Gn, pVax1-Gc, pVax1-N, pVax1-NSs, and pVax1-RdRp) twice at 3-week intervals. Control BALB/c mice (*n* = 4) were immunized with a control plasmid backbone (pVax1). **b** Timeline of vaccination, immune analysis, and vaccine efficacy testing for the first ferret study. Naïve ferrets (*n* = 8, >4 years old, V1-V8) were immunized via intradermal injection with SFTSV DNA vaccines three times at 2-week intervals. Naïve ferrets in the control group (*n* = 7, >4 years old, C1-C7) were immunized with the control plasmid backbone (pVax1). The vaccinated and control ferrets were challenged with a lethal intramuscular dose of SFTSV at 2 or 4 weeks after their last vaccination. Naïve ferrets (*n* = 3; >4 years old; V15-V17, V18-V20) were immunized with a total 200 µg or 40 µg of SFTSV DNA vaccines three times at 2-week intervals. Naïve ferrets (*n* = 3; >4 years old; V9-V11, V12-V14) were immunized with SFTSV DNA vaccines two times at a 2-week interval, or one time. After SFTSV challenge, all ferrets were monitored for clinical signs of infection. **c** Timeline of vaccination, immune analysis, and vaccine efficacy testing for the second ferret study. Naïve ferrets in the control group (*n* = 6, >4 years old, C8-C13) were immunized with the control plasmid backbone (pVax1). Another group of naïve ferrets (*n* = 4, >4 years old, V21-V24) were immunized with SFTSV DNA vaccines (pVax1-Gn and pVax1-Gc) three times at 2-week intervals. After three rounds of vaccination, sera were collected from these vaccinated ferrets (p-Vax1, *n* = 2, S1-S2; Gn/Gc, *n* = 2, S3-S4; N/NSs/RdRp, *n* = 2, S5-S6), and transferred via intraperitoneal injection into a third group of naïve ferrets (pVax1, *n* = 3, V25-V27; Gn/Gc, *n* = 4, V28-V31; N/NSs/RdRp, *n* = 3, V32-V34). One day after serum injection, or 2 weeks after the third vaccination, these groups of vaccinated, serum-transferred, and control ferrets were challenged with a lethal intramuscular dose of SFTSV, and then monitored for clinical signs of infection. For the N/NSs/RdRp, N, NSs, or RdRp-vaccine group, naïve ferrets (>4 years old; *n* = 3, V35-V38; *n* = 3, V39-V41; *n* = 3, V42-V44; *n* = 3, V45-V47) were immunized with SFTSV DNA vaccines encoding non-envelope proteins (pVax1-N, pVax1-NSs, and pVax1-RdRp) three times at 2-week intervals. At 2 weeks after the third vaccination, all ferrets were challenged with a lethal dose of SFTSV, and then monitored for clinical signs of infection.

Supplementary Figure 2

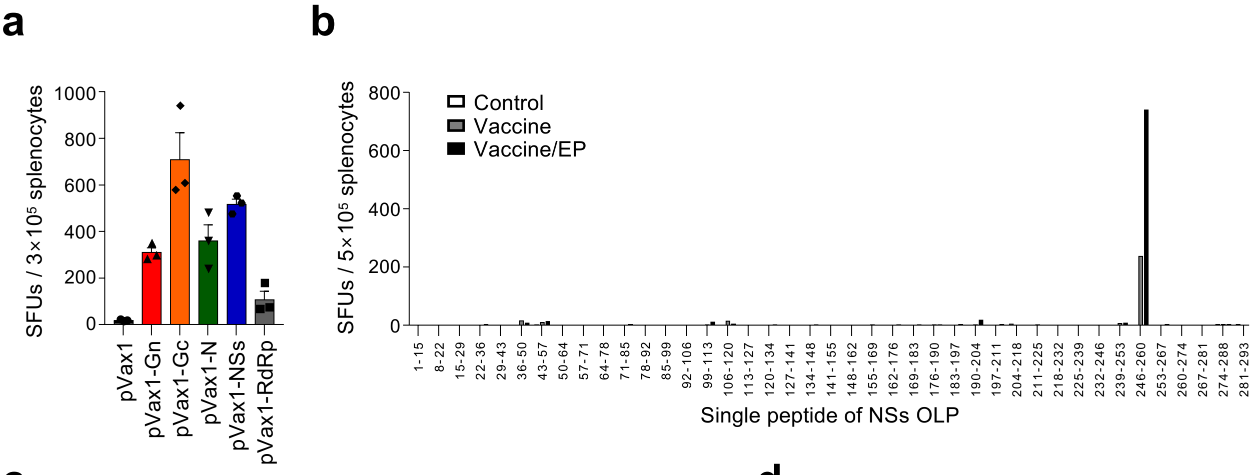

c

MHC restriction	Sequence	Position	1-log50k(aff)	Affinity(nM)
H-2L^d	YPYLMAHYL	247-255	44.10	0.650
H-2L^d	PYLMAHYL	248-255	1523.11	0.323
H-2L^d	GYPYLMAHYL	246-255	1858.72	0.304
H-2L^d	YPYLMAHYLN	247-256	1928.31	0.301
H-2L^d	YPYLMAHY	247-254	4032.43	0.233
H-2K^d	PYLMAHYL	248-255	214.21	0.504
H-2K^d	GYPYLMAHYL	246-255	262.12	0.250
H-2D^d	GYPYLMAHYL	246-255	3051.98	0.258
H-2D^d	GYPYLMAHY	246-254	5165.50	0.210
H-2D^d	GYPYLMAH	246-253	13613.23	0.120

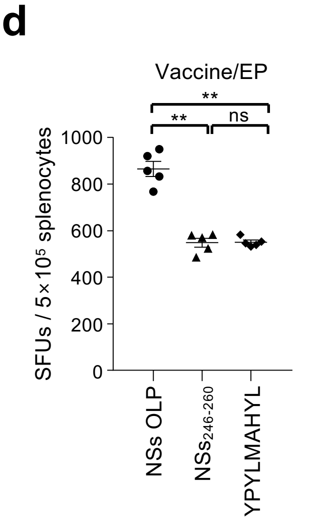

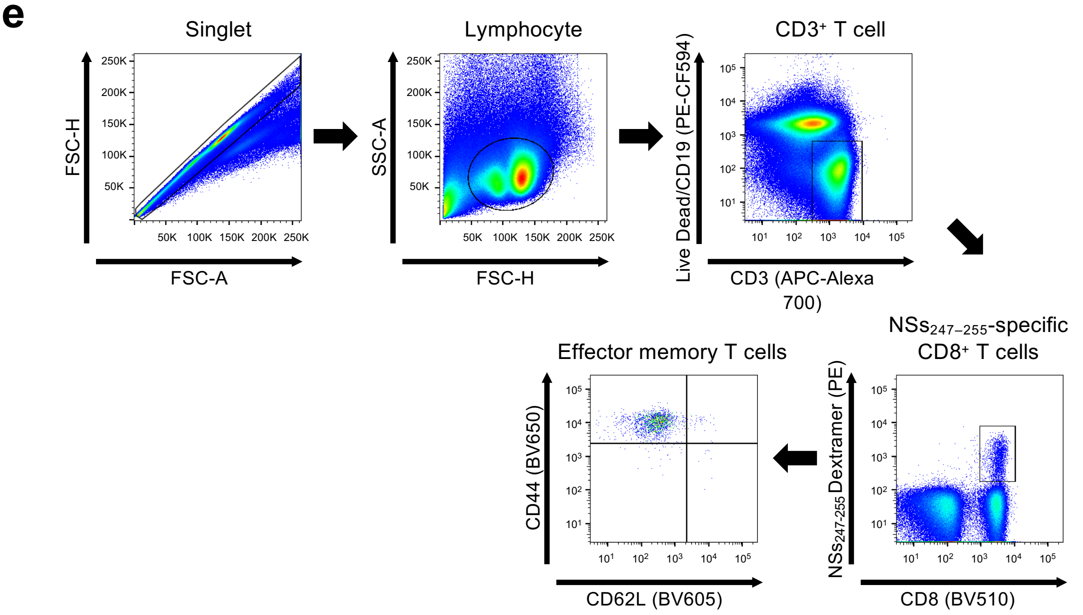

Supplementary Figure 2. Identification of H-2L^d-restricted immunodominant minimal epitope in SFTSV NSs. a T-cell immunogenicity of SFTSV DNA vaccines in BALB/c mice immunized with individual DNA vaccine encoding Gn (*n* = 3), Gc (*n* = 3), N (*n* = 3), NSs (*n* = 3), or RdRp (*n* = 3). **b** Five- to six-week-old female BALB/c mice were intramuscularly immunized twice at 2-week intervals with 40 μg of pVax1-NSs, followed by in vivo electroporation. IFN-γ ELISpot assays were performed by stimulating splenocytes from immunized mice with a single OLP from the SFTSV NS pool and an OLP (NSs$_{246–260}$) with high numbers of IFN-γ spots was identified. **c-d** The minimal epitope sequence (NSs$_{247–255}$, YPYLMAHYL) for high-affinity binding to mouse MHC class I, H-2L^d was identified using in silico NetMHC 4.0 analysis (c) and IFN-γ ELISPOT assays (d). **e** Gating strategy of phenotypic analysis of SFTSV NSs$_{247–255}$–specific CD8$^+$ T cells, which corresponds to FACS data panel in Figure 1e. Error bars indicate the mean ± s.e.m. Statistical significance was determined by two-tailed Mann-Whitney U test (d). *$^{**}p<0.01$.* Source data are provided as a Source Data file.

Supplementary Figure 3

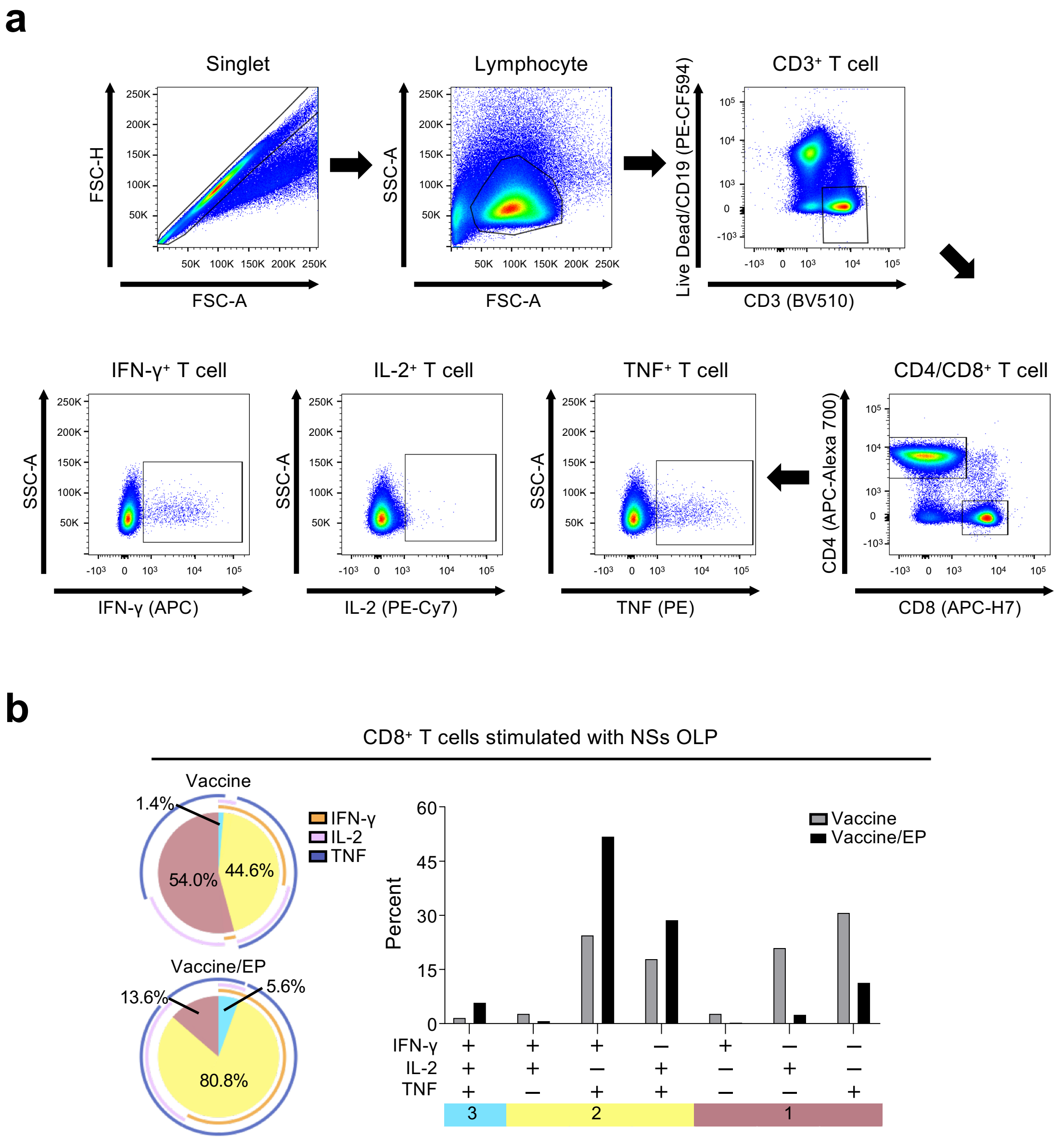

Supplementary Figure 3. CD8⁺ T cell polyfunctionality of SFTSV DNA vaccine. a Representative plot of ICS assay. **b** CD8⁺ T cell polyfunctionality of SFTSV DNA vaccine as analyzed based on every possible combination of functions. Pie graphs show the fraction of CD8⁺ T cells positive for a given number of functions. Source data are provided as a Source Data file.

Supplementary Figure 4

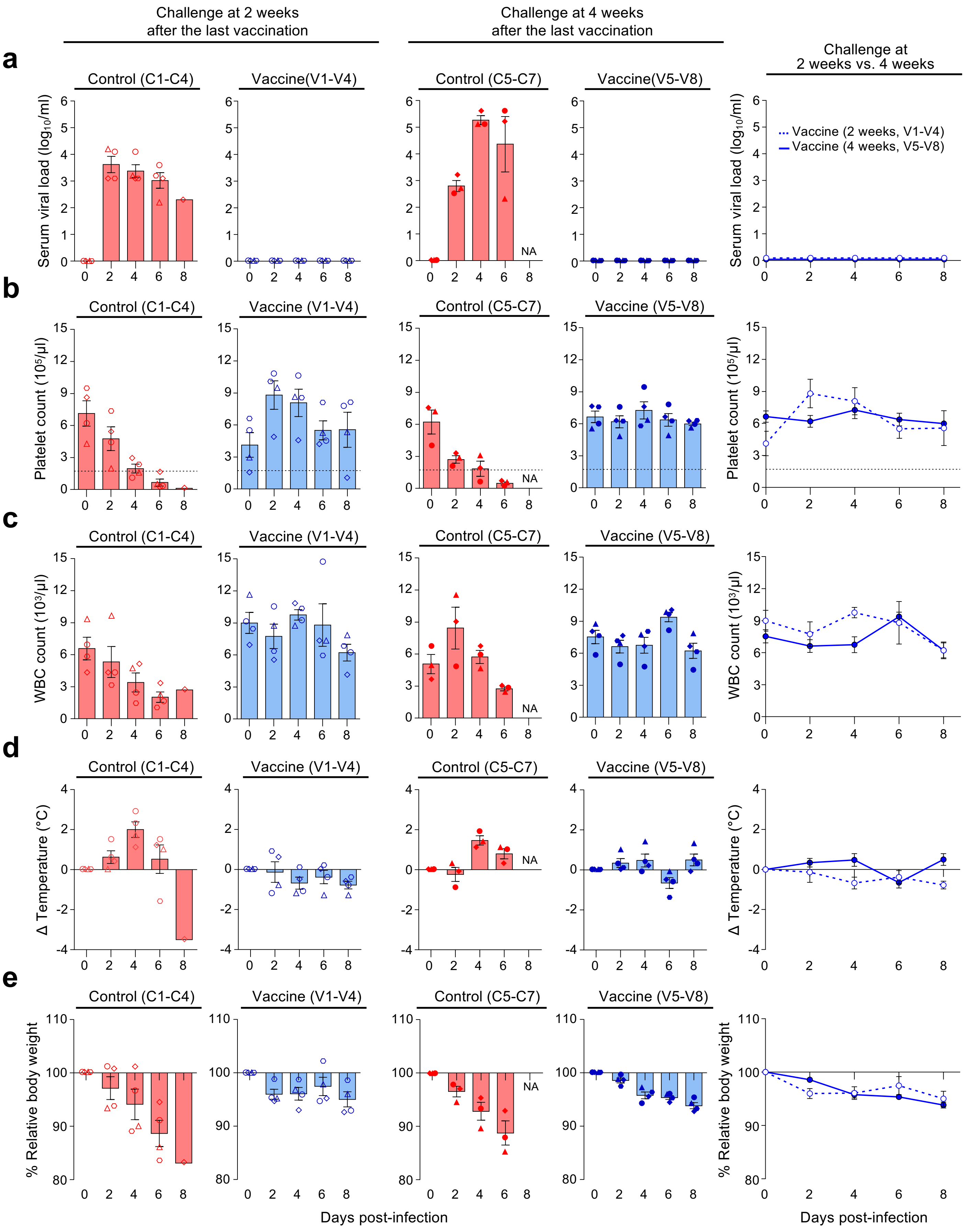

Supplementary Figure 4. Clinical signs in ferrets challenged at 2 or 4 weeks after their last vaccination. **a** Circulating viral titers in ferrets that were challenged at 2 weeks (open symbols) or 4 weeks (closed symbols) after their third vaccination. Blood was collected from ferrets every other day, and viral copy numbers were determined by real-time PCR. **b** Platelet counts of the vaccinated (blue bars, symbols and line) and non-vaccinated ferrets (red bars and symbols) after SFTSV challenge. The normal platelet count range in ferrets is 171.7–1280.6 × 10^3 per µL. Dashed lines indicate the normal platelet count values. **c–e** White blood cell counts (c), temperature (d), and relative weight (e) of vaccinated (blue bars, symbols and line) and non-vaccinated ferrets (red bars and symbols) after SFTSV challenge Error bars indicate the mean ± s.e.m. NA, not applicable mainly due to the deaths of ferrets after challenge. Source data are provided as a Source Data file.

Supplementary Figure 5

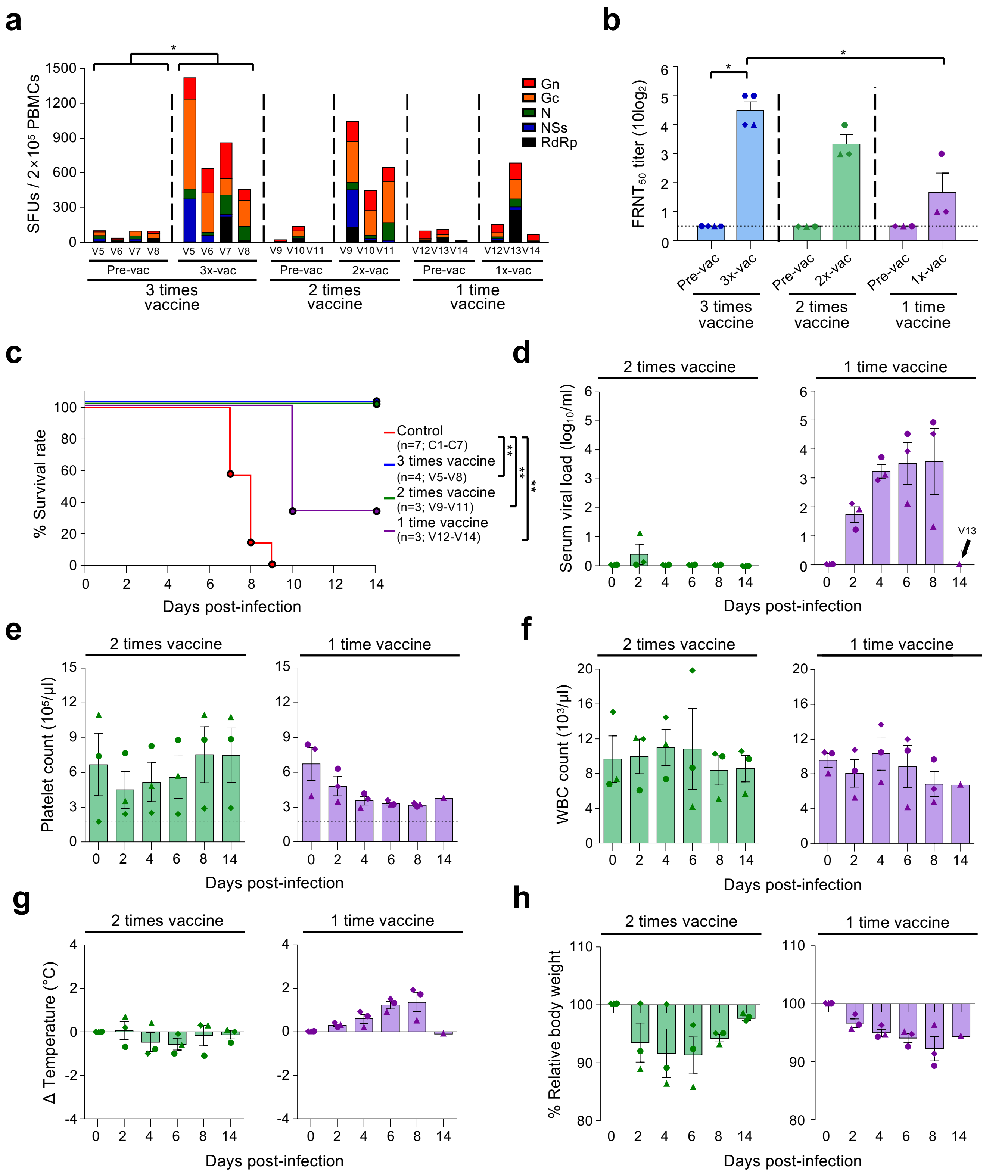

Supplementary Figure 5. Immunogenicity and protection efficacy after vaccination once or twice. a T-cell immunogenicity of SFTSV DNA vaccines in ferrets. IFN-γ ELISpot assays were performed to detect antigen-specific IFN-γ-producing T cells by stimulating peripheral blood mononuclear cells (PBMCs) from vaccinated ferrets with OLP pools. Data represent the average number of SFUs per 2×10^5 PBMCs. **b** Neutralizing antibody response to SFTSV CB1/2014 strain (genotype B) generated by DNA vaccines in ferrets. The amount of neutralizing antibody against SFTSV was determined based on $FRNT_{50}$. **c** Survival of twice-vaccinated (*n* = 3, green line) and once-vaccinated ferrets (*n* = 3, purple line) after lethal SFTSV challenge. **d** Circulating viral titers of twice-vaccinated ferrets (green bars and symbols) and once-vaccinated ferrets (purple bars and symbols) after SFTSV challenge. **e** Platelet counts in twice-vaccinated ferrets (green bars and symbols) and once-vaccinated ferrets (purple bars and symbols) after SFTSV challenge. The normal platelet count range in ferrets is $171.7–1280.6 \times 10^3$ per µL. Dashed lines indicate the normal platelet count values. **f–h** White blood cell counts (f), temperature (g), and relative weight (h) of twice-vaccinated ferrets (green bars and symbols) and once-vaccinated ferrets (purple bars and symbols). Error bars indicate the mean ± s.e.m. Statistical significance was determined by two-tailed Mann-Whitney U test (a, b) or log-rank (Mantel-Cox) test (c). *$p<0.05$; **$p<0.01$. Source data are provided as a Source Data file.

Supplementary Figure 6

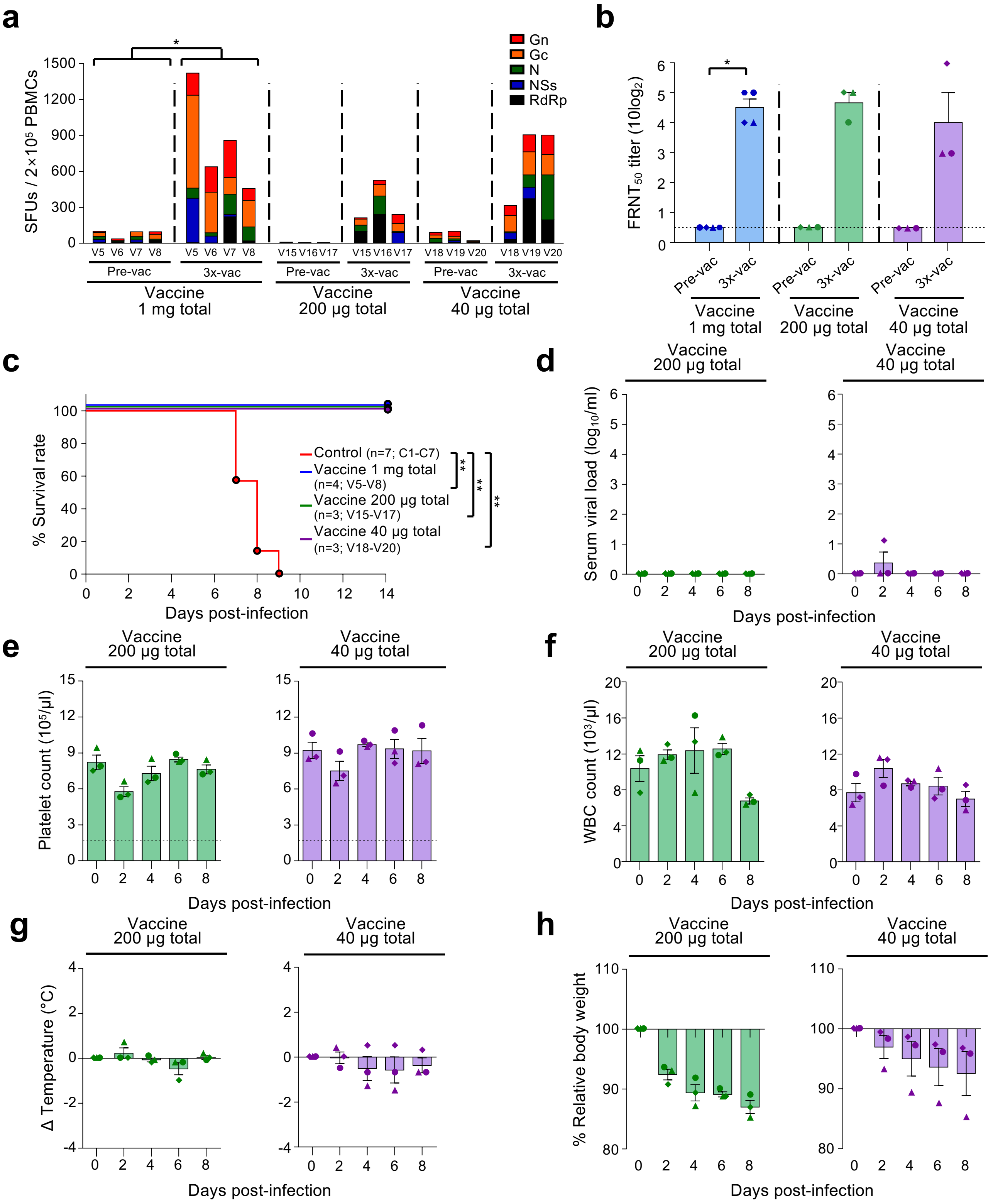

Supplementary Figure 6. Immunogenicity and protection efficacy after vaccination with a total of 200 µg or 40 µg of DNA vaccines. a T-cell immunogenicity of SFTSV DNA vaccines in ferrets. IFN-γ ELISpot assays were performed to detect antigen-specific IFN-γ-producing T cells by stimulating peripheral blood mononuclear cells (PBMCs) from vaccinated ferrets with OLP pools. Data represent the average number of SFUs per 2×10^5 PBMCs. **b** Neutralizing antibody response to SFTSV CB1/2014 strain (genotype B) generated by DNA vaccines in ferrets. The amount of neutralizing antibody against SFTSV was determined based on $FRNT_{50}$. **c** Survival after lethal SFTSV challenge among ferrets that were vaccinated with a total of 200 µg (n = 3, green line) or 40 µg (n = 3, purple line) of DNA vaccines. **d** Circulating viral titers of vaccinated ferrets after SFTSV challenge. **e** Platelet counts in vaccinated ferrets after SFTSV challenge. The normal platelet count range in ferrets is 171.7–1280.6 × 10^3 per µL. Dashed lines indicate the normal platelet count values. **f–h** White blood cell counts (f), temperature (g), and relative weight (h) of ferrets that were vaccinated with 200 µg (green bars and symbols) or 40 µg total DNA vaccines (purple bars and symbols). Error bars indicate the mean ± s.e.m. Statistical significance was determined by two-tailed Mann-Whitney U test (a, b) or log-rank (Mantel-Cox) test (c). *$p<0.05$; **$p<0.01$.* Source data are provided as a Source Data file.

Supplementary Figure 7

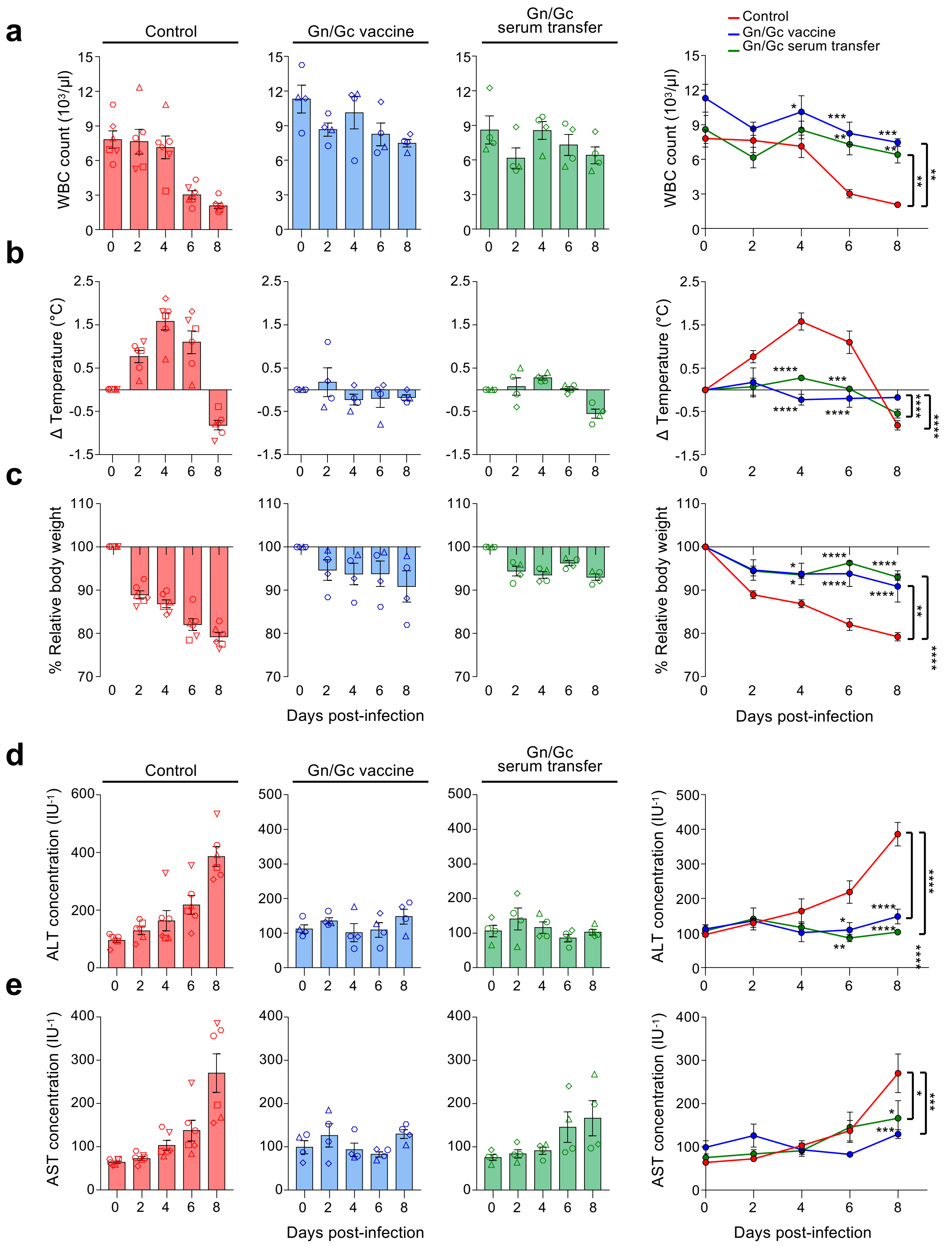

Supplementary Figure 7 (Cont.)

Supplementary Figure 7. Clinical signs in Gn/Gc-vaccinated and Gn/Gc serum-transferred ferrets after SFTSV challenge. a–c White blood cell counts (**a**), temperature (**b**), and relative weight (**c**) of Gn/Gc-vaccinated ferrets (*n* = 4, blue bars, symbols and line), non-vaccinated ferrets (*n* = 6, red bars, symbols and line), and Gn/Gc serum-transferred ferrets (*n* = 4, green bars, symbols and line) after SFTSV challenge. Data are shown as mean ± s.e.m. **d, e** ALT concentrations (**d**) and AST concentrations (**e**) in Gn/Gc-vaccinated ferrets (blue bars, symbols and line), non-vaccinated ferrets (red bars, symbols and line), and Gn/Gc serum-transferred ferrets (green bars, symbols and line) after SFTSV challenge. Asterisks indicate significance between vaccinated and control ferrets per dpi. Error bars indicate the mean ± s.e.m. Statistical significance was determined by two-way ANOVA test with Sidak correction (a–e). *$p<0.05$; **$p<0.01$; ***$p<0.001$; ****$p<0.0001$. Source data are provided as a Source Data file.

Supplementary Figure 8

Group	Ferret #	Vaccination		
Serum source for control serum transfer Gr (n=2)	S1-S2	pVax1	(1 mg)	→ collection of serum at day -1
Serum source for Gn/Gc serum transfer Gr (n=2)	S3-S4	pVax1-Gn<br>pVax1-Gc	(200 μg)<br>(200 μg)	→ collection of serum at day -1
Serum source for N/NSs/RdRp serum transfer Gr (n=2)	S5-S6	pVax1-N<br>pVax1-NSs<br>pVax1-RdRp	(200 μg)<br>(200 μg)<br>(200 μg)	→ collection of serum at day -1

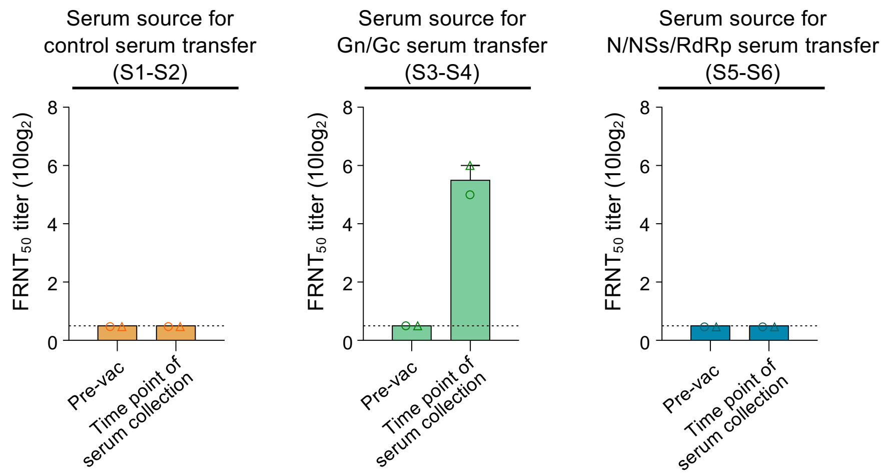

Supplementary Figure 8. Neutralizing antibody response in vaccinated ferrets for serum correction. Neutralizing antibody titers were evaluated by $FRNT_{50}$ at the time of serum collection from pVax1-vaccinated ferrets (*n* = 2, S1-S2, orange bars and symbols), Gn/Gc-vaccinated ferrets (*n* = 2, S3-S4, green bars and symbols), and N/NSs/RdRp-vaccinated ferrets (*n* = 2, S5-S6, blue bars and symbols). Error bars indicate the mean ± s.e.m. Source data are provided as a Source Data file.

Supplementary Figure 9

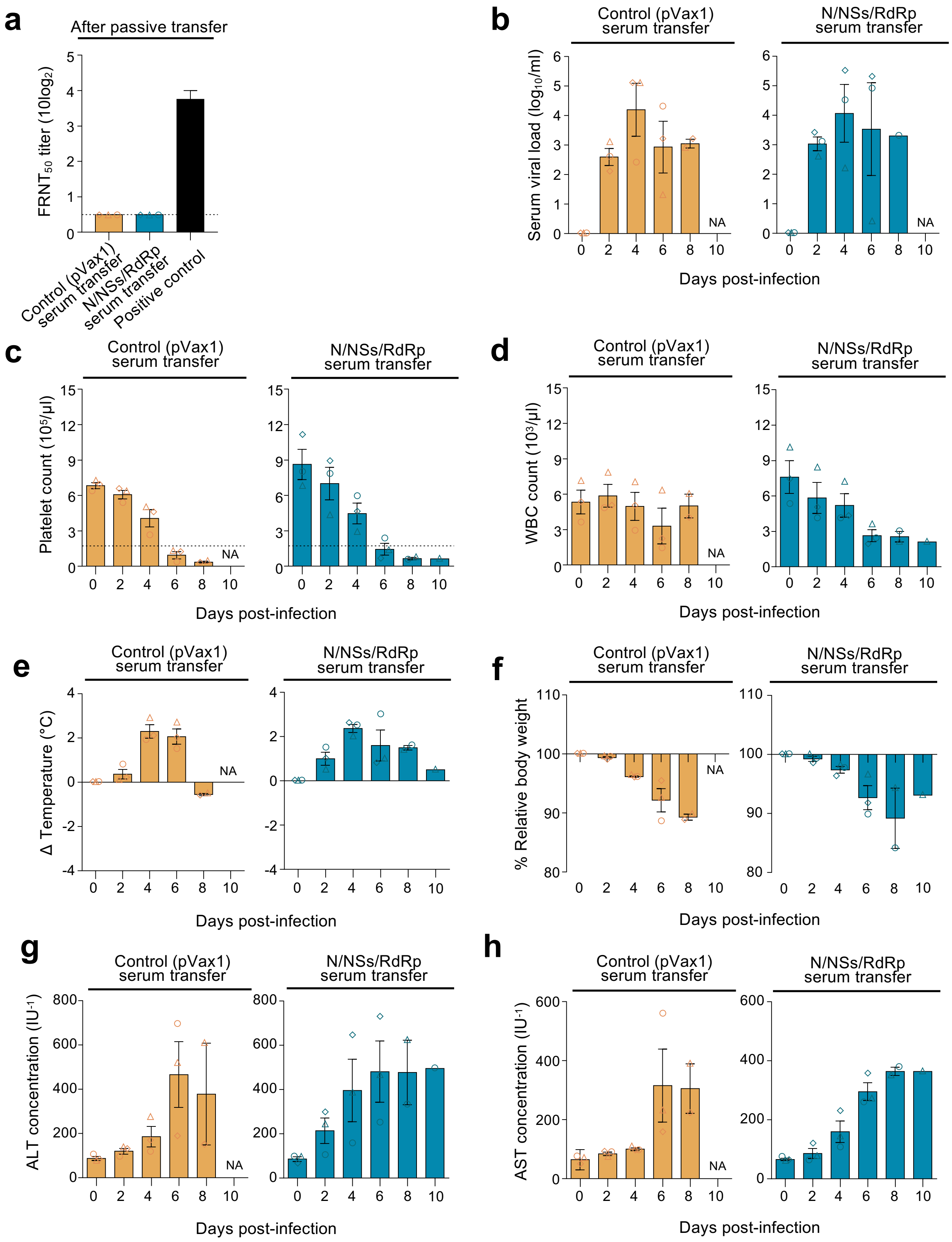

Supplementary Figure 9. Control and N/NSs/RdRp serum transfer groups were not protected from SFTSV challenge. a Neutralizing antibody response to SFTSV CB1/2014 strain (genotype B) generated by passive transfer in ferrets. The amount of neutralizing antibody against SFTSV was determined based on $FRNT_{50}$. **b** Circulating viral titers of the control (n = 3, orange bars and symbols) and N/NSs/RdRp serum-transferred ferrets (n = 3, blue bars and symbols) after SFTSV challenge. Blood was collected from ferrets every other day, and viral copy numbers were determined by real-time PCR. **c** Platelet counts of the control (orange bars and symbols) and N/NSs/RdRp serum-transferred ferrets (blue bars and symbols) after SFTSV challenge. The normal platelet count range in ferrets is 171.7–1280.6 × 10^3 per µL. Dashed lines indicate the normal platelet count values. **d–f** White blood cell counts (d), temperature (e), and relative weight (f) of control (orange bars and symbols) and N/NSs/RdRp serum-transferred ferrets (blue bars and symbols) after SFTSV challenge. **g, h** ALT concentrations (g) and AST concentrations (h) from control (orange bars and symbols) and N/NSs/RdRp serum-transferred ferrets (blue bars and symbols) after SFTSV challenge. Error bars indicate the mean ± s.e.m. NA, not applicable mainly due to the deaths of ferrets after challenge. Source data are provided as a Source Data file.

Supplementary Figure 10

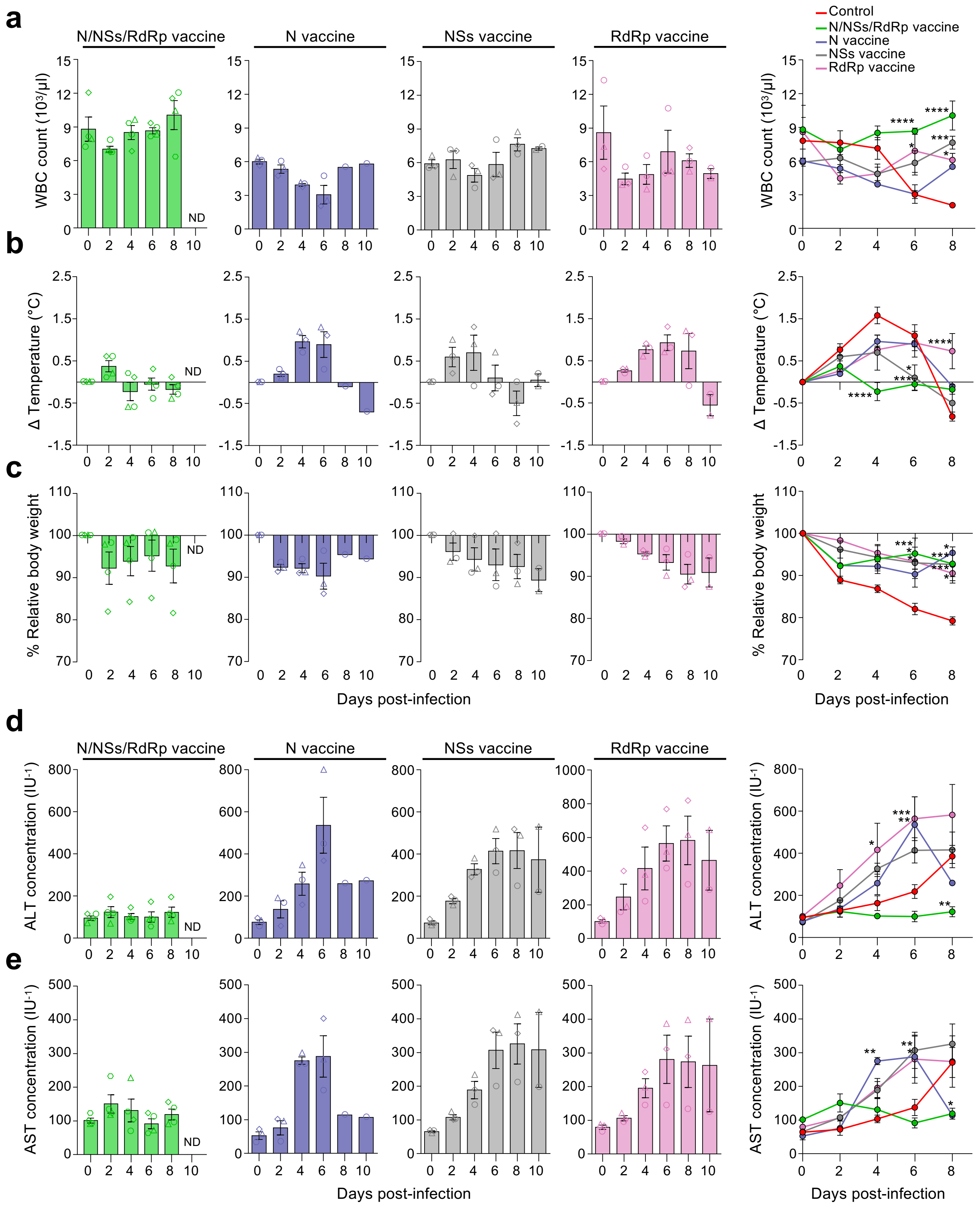

Supplementary Figure 10 (Cont.)

Supplementary Figure 10. Clinical signs of N/NSs/RdRp-, N-, NSs-, or RdRp-vaccinated ferrets after SFTSV challenge. a–c White blood cell counts (a), temperature (b), and relative weight (c) of N/NSs/RdRp- (*n* = 4, green bars, symbols and line), N- (*n* = 3, indigo bars, symbols and line), NSs- (*n* = 3, gray bars, symbols and line), or RdRp-vaccinated ferrets (*n* = 3, pink bars, symbols and line) and non-vaccinated ferrets (red line) after SFTSV challenge. **d, e** ALT concentrations (d) and AST concentrations (e) from N/NSs/RdRp- (green bars, symbols and line), N- (indigo bars, symbols and line), NSs- (gray bars, symbols and line), or RdRp-vaccinated ferrets (pink bars, symbols and line) and non-vaccinated ferrets (red line) after SFTSV challenge. Asterisks indicate significance between vaccinated and control ferrets per dpi. Error bars indicate the mean ± s.e.m. Statistical significance was determined by two-way ANOVA test with Sidak correction (a–e). ND, not determined due to the sample unavailability. *$p<0.05$; **$p<0.01$; ***$p<0.001$; ****$p<0.0001$. Source data are provided as a Source Data file.

Supplementary Table 1. A list of 31 SFTSV isolates to generate consensus sequences of SFTSV DNA vaccines

Strain	Origin	Year	Genotype	GenBank accession No. (L/M/S)
CB3	Korea	2016	B	KY789435, KY789438, KY789441
CB2	Korea	2015	A	KY789434, KY789437, KY789440
CB1	Korea	2014	B	KY789433, KY789436, KY789439
KASJH	Korea	2014	D	KP663731, KP663732, KP663733
KACNH3	Korea	2014	B	KP663743, KP663744, KP663745
KAGBH6	Korea	2014	B	KP663740, KP663741, KP663742
KAGBH5	Korea	2014	B	KP663737, KP663738, KP663739
KAGWH3	Korea	2014	B	KP663734, KP663735, KP663736
JZHSH-LWL	China	2014	B	KR017844, KR017863, KR017825
ZJZHSH-WRF	China	2014	B	KR017865, KR017864, KR017866
SPL003A	Japan	2014	B	AB817980, AB817988, AB817996
SPL035A	Japan	2014	B	AB817986, AB817994, AB818002
SPL010A	Japan	2014	B	AB817983, AB817991, AB817999
YG1	Japan	2014	B	AB817979, AB817987, AB817995
Gangwon	Korea	2012	F	KF358691, KF358692, KF358693
Zhao	China	2011	B	KF374682, KF374684, AF374683
Zhejiang/01	China	2011	B	KJ597825, KJ597824, KJ597823LA
HB156	China	2011	D	JQ733567, JQ733566, JQ733568
HN6	China	2011	F	HQ141595, HQ141596, HQ141597
JS2011-027	China	2011	B	KC505129, KC505130, KC505131
JS3	China	2010	A	HQ141601, HQ141602, HQ141603
JS4	China	2010	E	HQ141604, HQ141605, HQ141606
JS6	China	2010	A	HQ830169, HQ830170, HQ830171
JS2010-014	China	2010	F	JQ317171, JQ317170, JQ317169
JS2010-015	China	2010	F	JQ317172, JQ317173, JQ317174
JSD1	China	2010	F	JF267783, JF267784, JF267785
LN2	China	2010	F	HQ141607, HQ141608, HQ141608
HB29	China	2010	D	NC018136, NC018138, NC018137
SD4	China	2010	E	HM802202, HM802203, HM802204
AH15	China	2010	A	HQ141592, HQ141593, HQ141594
AH12	China	2010	F	HQ116417, HQ141590, HQ141591
