# Supplementary material for: Development of a SFTSV DNA vaccine that confers complete protection against lethal infection in ferrets
Source: Nat Commun. 2019 Aug 23;10:3836. doi: 10.1038/s41467-019-11815-4 (PMC6707330; doi:10.1038/s41467-019-11815-4)
Supplement: Supplementary file 3 — Reporting Summary [file 41467_2019_11815_MOESM3_ESM.pdf]

nature research

Corresponding author(s): Su-Hyung Park and Young Ki Choi

Last updated by author(s): Feb 6, 2019

Reporting Summary

Nature Research wishes to improve the reproducibility of the work that we publish. This form provides structure for consistency and transparency in reporting. For further information on Nature Research policies, see Authors & Referees and the Editorial Policy Checklist .

Statistics

For all statistical analyses, confirm that the following items are present in the figure legend, table legend, main text, or Methods section.

n/a	Confirmed	
☐	☒	The exact sample size (*n*) for each experimental group/condition, given as a discrete number and unit of measurement
☐	☒	A statement on whether measurements were taken from distinct samples or whether the same sample was measured repeatedly
☐	☒	The statistical test(s) used AND whether they are one- or two-sided<br>*Only common tests should be described solely by name; describe more complex techniques in the Methods section.*
☐	☒	A description of all covariates tested
☐	☒	A description of any assumptions or corrections, such as tests of normality and adjustment for multiple comparisons
☐	☒	A full description of the statistical parameters including central tendency (e.g. means) or other basic estimates (e.g. regression coefficient) AND variation (e.g. standard deviation) or associated estimates of uncertainty (e.g. confidence intervals)
☒	☐	For null hypothesis testing, the test statistic (e.g. *F*, *t*, *r*) with confidence intervals, effect sizes, degrees of freedom and *P* value noted<br>*Give P values as exact values whenever suitable.*
☒	☐	For Bayesian analysis, information on the choice of priors and Markov chain Monte Carlo settings
☒	☐	For hierarchical and complex designs, identification of the appropriate level for tests and full reporting of outcomes
☒	☐	Estimates of effect sizes (e.g. Cohen's *d*, Pearson's *r*), indicating how they were calculated

Our web collection on statistics for biologists contains articles on many of the points above.

Software and code

Policy information about availability of computer code

Data collection	No commercial or open source was collected and used. All data was collected through experimental findings.
Data analysis	All statistical analyses were performed using GraphPad Prism version 8. P values less than 0.05 (95% confidence interval) were considered significant. For all FACS analyses, Flowjo version 10 was used.

For manuscripts utilizing custom algorithms or software that are central to the research but not yet described in published literature, software must be made available to editors/reviewers. We strongly encourage code deposition in a community repository (e.g. GitHub). See the Nature Research guidelines for submitting code & software for further information.

Data

Policy information about availability of data

All manuscripts must include a data availability statement. This statement should provide the following information, where applicable:

- Accession codes, unique identifiers, or web links for publicly available datasets
- A list of figures that have associated raw data
- A description of any restrictions on data availability

The data that support the findings of this study are available from the corresponding author S.H.P. upon reasonable request. The source data underlying Figs 1b, 1d–f, 2b–h, 3b–g and 4a–e and Supplementary Figs 2a–b, 2d, 3b, 4a–e, 5a–h, 6a–h, 7a–e, 8, 9a–h and 10a–e are provided as a Source Data file.

Field-specific reporting

Please select the one below that is the best fit for your research. If you are not sure, read the appropriate sections before making your selection.

Life sciences study design

All studies must disclose on these points even when the disclosure is negative.

Sample size	No statistical method was used to predetermine sample size. Sample sizes were selected based on previous experience to obtain statistical significance and reproducibility.
Data exclusions	No data has been excluded.
Replication	All experiments for investigating vaccine-induced immunity and clinical symptoms after SFTSV challenge were performed with at least triplicates. All the experimental findings were reliably reproduced.
Randomization	For all animal studies, mice and ferrets were randomly assigned to each group.
Blinding	The investigators were not blinded to group allocation during DNA vaccination and collection of specimens from animals. However, the investigators were blinded to group allocation during data acquisition/analysis such as the measurements of survival rates, serum viral titers, platelet counts, body weights and body temperatures after lethal SFTSV challenge.

Reporting for specific materials, systems and methods

We require information from authors about some types of materials, experimental systems and methods used in many studies. Here, indicate whether each material, system or method listed is relevant to your study. If you are not sure if a list item applies to your research, read the appropriate section before selecting a response.

Materials & experimental systems

n/a	Involved in the study	
☐	☒	Antibodies
☐	☒	Eukaryotic cell lines
☒	☐	Palaeontology
☐	☒	Animals and other organisms
☒	☐	Human research participants
☒	☐	Clinical data

Methods

n/a	Involved in the study	
☒	☐	ChIP-seq
☐	☒	Flow cytometry
☒	☐	MRI-based neuroimaging

Antibodies

Antibodies used	For in vitro neutralizing assay, in-house generated NP monoclonal antibody as a primary antibody and HRP-conjugated anti mouse Ig G antibody as a secondary antibody (cat #115-035-146, lot # 126768, Jackson ImmunoResearch) were used.<br>For FACS analysis (ICS assay, dextramer staining), anti-CD19–PE-CF594 (1D3), anti-CD8–BV510 (53-6.7), anti-CD8–APC-H7 (53-6.7), anti-CD4–Alexa Flour 700 (RM4-5), anti-CD3–BV510 (145-2C11), anti-TNF–PE (MP6-XT22), anti-IL-2–PE-Cy7 (JES6-5H4), anti-CD62L–BV605 (MEL-14; all from BD Biosciences), anti-CD44–BV650 (IM7), anti-CD3–Alexa Fluor 700 (500A2; all from eBioscience), and anti-IFN-γ–APC (XMG1.2; from BioLegend) were purchased and used in this study.
Validation	Primary NP antibody was made in our laboratory and was validated through western blotting and immuno fluorescence assay.

Eukaryotic cell lines

Policy information about cell lines

Cell line source(s)	Vero E6 cells (ATCC No. CRL-1586; American Type Culture Collection, Manassas, VA) were cultured in Dulbecco's Modified Eagle Medium (DMEM; Gibco, Grand Island, NY) containing 2% fetal bovine serum (FBS; Gibco) with penicillin (100 U/ml) and streptomycin (100 µg/ml; P/S, Gibco) placed in 37°C incubator supplemented with 5% CO2.
Authentication	Vero E6 cells were purchased from ATCC (No. CRL-1586; American Type Culture Collection, Manassas, VA)
Mycoplasma contamination	All cells were mycoplasma-free.
Commonly misidentified lines (See ICLAC register)	No commonly misidentified cell lines were used.

Animals and other organisms

Policy information about studies involving animals; ARRIVE guidelines recommended for reporting animal research

Laboratory animals	Five to six-week-old female BALB/c mice were purchased from DBL (Republic of Korea), and used for DNA vaccination. Aged ferrets (total n = 70, > 4 years old) were used for vaccine studies. All mouse experiment protocols were approved by the Animal Care Committee of Korea Advanced Institute of Science and Technology. All ferret experiments were approved by the Medical Research Institute, a member of Laboratory Animal Research Center of Chungbuk National University (LARC) (approval number: CBNUA-986-16-01), and conducted in BSL3 facility (KCDC-14-3-07).
Wild animals	No wild animal was used in this study.
Field-collected samples	This study did not involve samples collected from the field.
Ethics oversight	For all experiments using mice and ferrets, we have complied with all relevant ethical regulations for animal testing and research. Mouse care and experimental procedures were performed with the approval from the Animal Care Committee of Korea Advanced Institute of Science and Technology. All ferret experiments were approved by the Medical Research Institute, a member of Laboratory Animal Research Center of Chungbuk National University (LARC) (approval number: CBNUA-986-16-01) and were conducted in strict accordance and adherence to relevant policies regarding animal handling as mandated under the Guidelines for Animal Use and Care of the Korea Center for Disease Control (K-CDC).

Note that full information on the approval of the study protocol must also be provided in the manuscript.

Flow Cytometry

Plots

Confirm that:

- [x] The axis labels state the marker and fluorochrome used (e.g. CD4-FITC).
- [x] The axis scales are clearly visible. Include numbers along axes only for bottom left plot of group (a 'group' is an analysis of identical markers).
- [x] All plots are contour plots with outliers or pseudocolor plots.
- [x] A numerical value for number of cells or percentage (with statistics) is provided.

Methodology

Sample preparation	The preparation of each sample is described in the methods section.
Instrument	BD LSR II was used in this study.
Software	Flowjo version 10 was used for data analyses.
Cell population abundance	Not applicable
Gating strategy	The population of interest was gated on FSC-A vs FSC-H to exclude doublets, and then gated on FSC-A vs SSC-A to identify lymphocytes population. Dead cells were excluded by Live/Dead Fixable Red Dead Cell stain kit (Invitrogen). Cytokine-secreting T cells were identified based on cell populations of unstimulated controls and isotype controls. Detailed protocols were described in the methods section. Figures exemplifying the gating strategy are added in the Supplementary figures.

- [x] Tick this box to confirm that a figure exemplifying the gating strategy is provided in the Supplementary Information.
